# Two *SOX11* variants cause Coffin–Siris syndrome with a new feature of sensorineural hearing loss

**DOI:** 10.1002/ajmg.a.63011

**Published:** 2022-11-11

**Authors:** Qiuquan Wang, Jie Wu, Jinyuan Yang, Shasha Huang, Yongyi Yuan, Pu Dai

**Affiliations:** ^1^ College of Otolaryngology Head and Neck Surgery Chinese PLA General Hospital, Chinese PLA Medical School Beijing China; ^2^ National Clinical Research Center for Otolaryngologic Diseases Beijing China; ^3^ State Key Lab of Hearing Science, Ministry of Education Beijing China; ^4^ Beijing Key Lab of hearing loss Prevention and Treatment Beijing China

**Keywords:** Coffin–Siris syndrome, inner ear malformation, sensorineural hearing loss, *SOX11*

## Abstract

Coffin‐Siris syndrome (CSS, OMIM#135900) is a rare congenital disorder associated with neurodevelopmental and dysmorphic features. The primary cause of CSS is pathogenic variants in any of 9 BAF chromatin‐remodeling complex encoding genes or the genes *SOX11* and *PHF6*. Herein, we performed whole‐exome sequencing (WES) and a series of analyses of growth‐related, auditory, and radiological findings in two probands with syndromic sensorineural hearing loss and inner ear malformations who exhibited distinctive facial features, intellectual disability, growth retardation, and fifth finger malformation. Two de novo variants in the *SOX11* gene (c.148A>C:p.Lys50Asn; c.811_814del:p.Asn271Serfs*10) were detected in these probands and were identified as pathogenic variants as per ACMG guidelines. These probands were diagnosed as having CSS based upon clinical and genetic findings. This is the first report of CSS caused by variants in *SOX11* gene in Chinese individuals. Deleterious *SOX11* variants can result in sensorineural hearing loss with inner ear malformation, potentially extending the array of phenotypes associated with these pathogenic variants. We suggest that both genetic and clinical findings be considered when diagnosing syndromic hearing loss.

**Abbreviations**
40 Hz AERP: 40 Hz auditory event‐related potential
ABR: auditory brainstem response
ACMG: American College of Medical Genetics and Genomics
ASSR: auditory steady‐state evoked potential
BAF: BRM‐associated factor
BFLS: Borjeson–Forssman–Lehmann syndrome
CSS: Coffin–Siris syndrome
CT: computed tomography
DPOAE: distortion product otoacoustic emission
DQ: development quotient
EEG: electroencephalogram
EXAC: Exome Aggregation Consortium
HGMD: Human Gene Mutation Database
HMG box: high mobility domain
MRI: magnetic resonance imaging
NCBRS: Nicolaides–Baraitser syndrome
OFC: occipitofrontal circumference
PCR: polymerase chain reaction
PGD: preimplantation genetic diagnosis
VDW: van der Waals
WES: whole‐exome sequencing

## 1 BACKGROUND

Coffin–Siris syndrome (CSS, OMIM#135900) is a rare congenital syndrome associated with multiple malformations that was first described in 1970 (Coffin & Siris, 1970), with affected patients often exhibiting developmental delays, a coarse facial appearance, hirsutism, and hypoplastic or absent fifth distal phalanges, which historically represent the minimum findings for CSS diagnosis (Fleck et al., 2001). This condition can also be associated with other malformations of the cardiac, gastrointestinal, genitourinary, or central nervous systems, and some patients may present with feeding difficulties, slow growth, and ophthalmic abnormalities. Owing to phenotypic variability, however, CSS can be challenging to diagnose (Fleck et al., 2001; Schrier Vergano et al., 1993), and can be challenging to differentiate from phenotypically similar syndromes including Nicolaides–Baraitser syndrome (NCBRS, OMIM#601358) and Borjeson–Forssman–Lehmann syndrome (BFLS, OMIM#301900).

CSS is usually sporadic, was refractory to conventional linkage but now know to be due to mostly de novo AD variants in several genes, including CSS1 caused by variants in *ARID1B* (OMIM#135900), CSS2 (ARID1A, OMIM#614607), CSS3 (SMARCB1, OMIM#614608), CSS4 (SMARCA4, OMIM#614609), CSS5 (SMARCE1, OMIM#616938), CSS6 (ARID2, OMIM#617808), CSS7 (DPF2, OMIM#601671), CSS8 (SMARCC2, OMIM#601734), CSS11 (SMARCD1, OMIM#618779). All of these causative genes encode subunits of the BRG1‐/BRM‐associated factor (BAF) chromatin remodeling complex (mSWI/SNF complex), and contribute 60% of the pathogenic gene variants in CSS (Schrier Vergano et al., 1993). In addition, CSS9 is caused by variants in *SOX11* (OMIM#615866), and CSS can also be caused by variants in *PHF6* (OMIM#300414), with both of these genes being involved in the process of chromatin remodeling. Although <300 cases of CSS patients have been reported, genotype–phenotype correlational studies have observed dramatic variations in disease frequency and severity as a function of the causative gene variants (Bramswig et al., 2017; Miyake et al., 2014; Tsurusaki, Okamoto, et al., 2014; Vasileiou et al., 2018; Zweier et al., 2015).

Only a few studies to date have reported on cases of CSS caused by *SOX11* variants, and no prior studies have reported *SOX11* related CSS in Chinese individuals. Herein, we report two unrelated Chinese patients exhibiting sporadic CSS associated with novel variants in the *SOX11* gene. Both patients suffered from sensorineural hearing loss with inner ear malformations, which is a phenotype that has not previously been reported in CSS patients with *SOX11* variants.

## 2 METHODS

### 2.1 Patients

Both subjects of this study were males with hearing loss (proband 1: 3‐years‐old; proband 2: 10‐months‐old) who underwent evaluation in the Genetic Testing Center for deafness, Chinese PLA General Hospital. Physical and audiological examinations, radiological assessments, and growth and developmental evaluations were recommended. Parents provided lineage details and other clinical information about these two probands, in addition to providing written informed consent for study participation. This study was approved by the Ethics Committee of People's Liberation Army General Hospital (reference number S2016‐120‐02), and was consistent with the Declaration of Helsinki.

### 2.2 Whole‐exome sequencing

A 2 ml sample of peripheral venous blood was collected from each proband and their family members after having received appropriate consent, using EDTA as an anticoagulant. Genomic DNA extraction was conducted with a QIAam Whole Blood DNA Extraction Kit (Qiagen, Germany) based upon provided instructions. A whole‐genome library was then prepared, and exon regions were captured with the GenCap liquid phase technology. Testing for proband 1 was performed by Mygenostics (Beijing, China), while testing for proband 2 was performed by Grandomics (Beijing, China). In both cases, captured region sequencing was performed with a second‐generation Illumina Nextseq500 instrument and a 150 bp read length. Adapters and low‐quality reads were then excluded, and cleaned reads were mapped to the UCSC hg19 human reference genome with BWA.

### 2.3 In silico assessment of variant pathogenicity

Multiple databases were queried for sequence variants, including the gnomAD, the Exome Aggregation Consortium (EXAC), 1000 Genome, and Human Gene Mutation Database (HGMD) databases. The evolutionary conservation of mutated amino acids was assessed by aligning orthologues in the HomoloGene database and by using the REVEL, MutationTaster, PolyPhen2, PROVEAN, and SIFT predictive programs to assess the pathogenicity of variants. Protein structure and variant impacts thereupon were assessed using mCSM‐PPI2 and Swiss‐model to, respectively, construct the dimensional models of wild‐type and mutant‐type molecules. We additionally predicted and calculated the change of affinity (ΔΔGAffinity) for the indicated variants. Lastly, variant pathogenicity was interpreted as per the American College of Medical Genetics and Genomics (ACMG) guidelines.

### 2.4 Sanger sequencing

Sanger sequencing was performed to confirm all variants. For proband 1, WES was performed for parents from the proband and his parents, while his siblings were analyzed via Sanger sequencing. Segregation analysis was performed for proband 2 and his parents.

Primers were synthesized according to the DNA fragments that were to be sequenced and were amplified by polymerase chain reaction (PCR) and sequenced via Sanger sequencing with an ABI3730xl sequencer (Applied Biosystems, USA). Results were compared with the reference sequence using the “Mutation Surveyor” software.

## 3 RESULTS

### 3.1 Clinical presentation of proband 1

Proband 1 is the third child of unrelated healthy parents. He was born by cesarean section at 39 weeks, with a weight of 3.3 kg (−0.05SD), a length of 50 cm (−0.5SD), and an occipitofrontal circumference (OFC) of 36.4 cm (+1.6SD).

Despite considerable efforts to adjust feeding patterns accordingly, proband 1 exhibited slow growth and development. At 22 months of age, a total amino acid examination revealed decreased ornithine and threonine levels consistent with nutritional disorders. The proband was able to sit on his own at 18 years of age, and was able to crawl on his stomach at 2 years and 5 months of age. At this time, the boy was 88 cm (−1.4SD) in length, 10.7 kg (−2.2SD) in weight, and had a head circumference of 45 cm(‐3SD). While his chronological age was 1 year and 10 months, his bone age was consistent with that of the child of 12 months of age. Muscular hypotonia was additionally noted. In addition, his external genitalia was relatively small.

Neuropsychological developmental testing was conducted at 6.4 months of age, at which time the proband exhibited a developmental quotient (DQ) score of 56, and his mental age was 3.8 months. These results indicated that his growth and intellectual development were moderately delayed.

**FIGURE 1:**
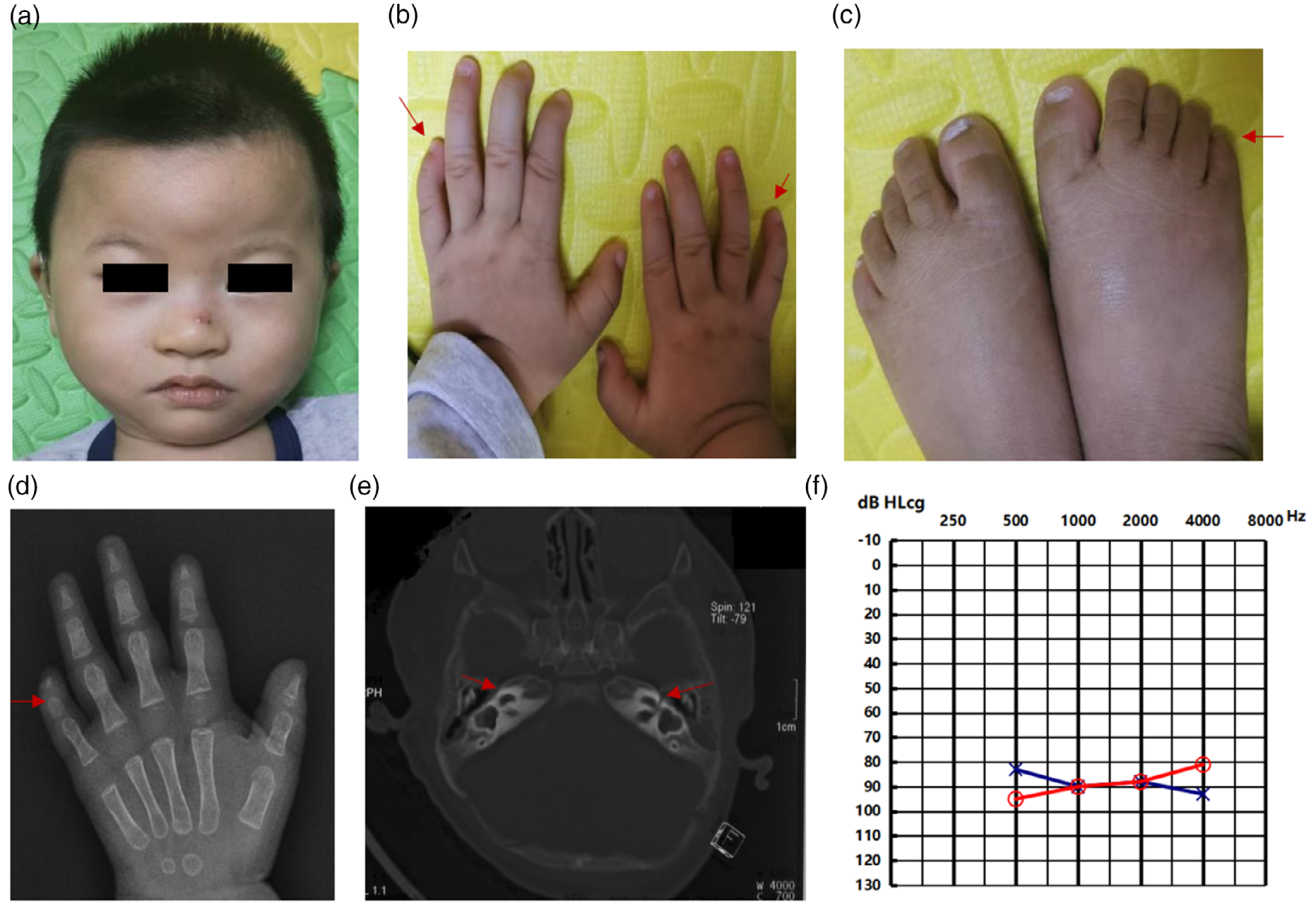
The phenotypic characteristics of proband 1. (a) The proband exhibited high anterior hairline, broad forehead with slightly flat zygomatic, low‐set ears with auricles that were turned backward, short nose with depressed nasal bridge, short columella with thick ala nasi, long and protruding philtrum, thin upper lip, and thick lower lip with teeth that were somewhat sparse. (b) The proband exhibited bilateral clinodactyly of the fifth finger with hypoplasia of the distal finger phalanx. (c) Feet demonstrating hypoplastic nails, with clinodactyly of the fifth toes. (d) X‐ray images of the left hand clearly showed hypoplastic distal phalanges of the fifth finger. (e) CT scans of the temporal bone revealed that the internal auditory canal was narrow on both sides of the inner ear, and the bilateral vestibular dilatation was cystic. (f) ASSR audiograms revealed showed severe to profound sensorineural hearing loss. Blue line—left ear, red line—right ear.

The proband did not pass neonatal hearing screening, and computed tomography (CT) scans of the temporal bone revealed that the internal auditory canal was narrow on both sides of the inner ear (Figure 1e). Auditory steady‐state evoked potential (ASSR) tests revealed profound sensorineural hearing loss in the right ear and severe sensorineural hearing loss in the left ear (Figure 1f). Auditory brainstem response (ABR) threshold examinations and 40 Hz auditory event‐related potentials exhibited 80 dB nHL in the left ear and no response in the right ear, with no meaningful distortion product otoacoustic emission (DPOAE) in either ear at all tested frequencies.

Clinical characters of the facial, musculoskeletal and other features of the proband 1 were compiled in Figure 1 and Table S1.

### 3.2 Clinical presentation of proband 2

Proband 2 is a 2‐year‐old boy born at 40.5 weeks of gestation to healthy parents after an uneventful pregnancy by vaginal delivery. He weighed 3.95 kg (+1.5SD) at birth, was 55 cm (+2.6SD) long, and his OFC was not recorded. Prenatal four‐dimensional ultrasounds reveal absent nasal bone.

The proband exhibited poor digestion. At 1‐month of age, his OFC was 38 cm (+1.8SD), and he was 56 cm (+0.5SD) long. At 28.3 months of age, he weighed 11.5 kg (−1.6SD), was 92 cm (−0.3SD) in height, and had an OFC of 49 cm (−0.07SD). He exhibited delayed development, and was unable to sit without support or to crawl at 9 months of age. Neuropsychological testing revealed moderate delays in growth of intellectual development. His overall DQ score was 59, and mental age was 16.7 months. X‐ray images revealed his bone age to be <2 years old when he was 2 years and 8 months old. The proband experienced seizures when he was 2 months old, and neurological examinations revealed appendicular hypotonia. Moreover, he has a history of recurrent respiratory tract infections.

**FIGURE 2:**
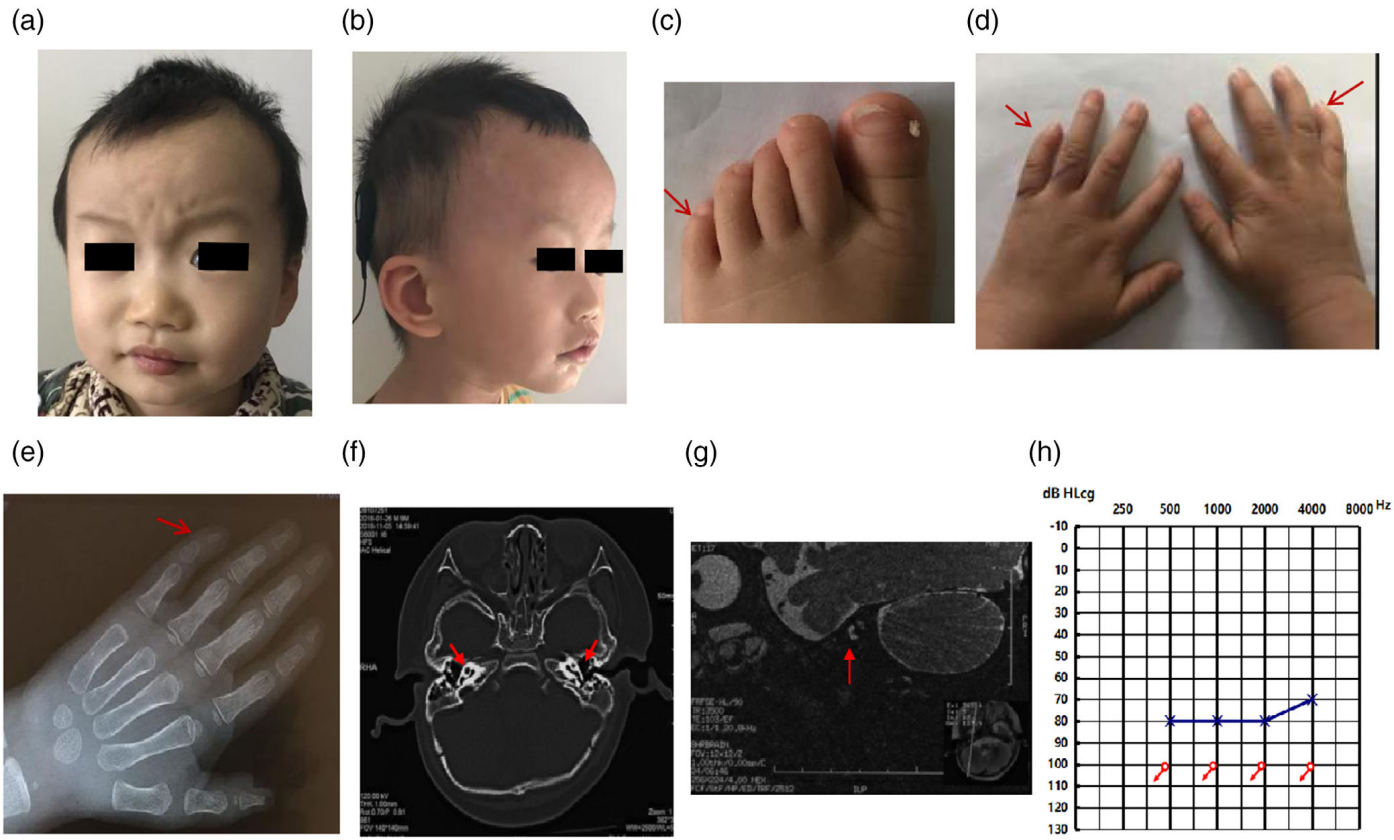
The phenotypic characteristics of proband 2. (a,b) The proband exhibited multiple unusual facial features, including high frontal hairline, sparse bilateral temporal hair, arched eyebrows, bulging forehead, wide mouth with thick lips, smooth philtrum, depressed nasal bridge, and short columella. His ears were low‐set ears with rotated backwards (c) The proband's feet exhibited hypoplastic nails, with clinodactyly of the fifth toe. (d) Bilateral clinodactyly of the fifth fingers with hypoplasia of the distal finger phalanx. (e) X‐ray image of the left hand revealed hypoplastic distal phalanges of the fifth finger. (f) A CT scan of the temporal bone revealed an incomplete partition type II cochlear malformation, with the cystic fusion of the apical turns and the middle circle. (g) Inner ear magnetic resonance hydrography failed to clearly resolve the cochlear nerve. (h) An ASSR audiogram of the proband exhibited profound sensorineural hearing loss. Blue line—left ear, red line—right ear.

The proband failed his neonatal hearing screening, and was diagnosed with bilateral profound sensorineural hearing loss when he was 10 months old by ASSR (Figure 2h), ABR, DPOAE, and 40 Hz auditory event‐related potential (40 Hz AERP). CT scans of his temporal bone revealed inner ear malformations including the cystic fusion of the apical turns and the middle circle, which was consistent with incomplete partition type II (Figure 2f). Inner ear magnetic resonance hydrography also failed to clearly display the cochlear nerve (Figure 2g), consistent with cochlear nerve dysplasia.

Clinical characters of the facial, musculoskeletal and other features of the proband 2 were compiled in Figure 2 and Table S1.

### 3.3 Molecular diagnosis

We identified two de novo *SOX11* variants in these two probands via WES—c.148A>C (p.Lys50Asn) in proband 1 and c.811_814del (p.Asn271Serfs*10) in proband 2. In addition to these gene variants, we did not detect any other causative variants in other known genes associated with hearing loss. As such, we excluded other hearing loss‐related genes as potential pathogenic variants in these patients. These variants have not previously been identified in Chinese CSS patients.

#### 3.3.1 In silico analysis of the c.148A>C (p.Lys50Asn) variant

The heterozygous c.148A>C:p.Lys50As variant was confirmed to be a de novo variant through the sanger sequencing.

This variant has not previously been recorded in public databases (gnomAD, ClinVar Database, HGMD, EXAC), nor have there been any prior literature reports regarding the clinical relevance of this variant. Hemple et al. previously described the same p.Lys50Asn protein variant caused by SOX11:c.150G>C (Hempel et al., 2016). This variant is associated with the substitution of an asparagine for a lysine (p.Lys50Asn) at a highly conserved residue across species which is located in the high mobility (HMG) box domain of the protein (Figure S1a,b). Like Hemple et al., we constructed a 3D model of Lys50Asn using mCSM‐PPI2, in addition, we also calculated the affinity change of p. Lys50Asn and found that it was reduced, with a ΔΔGAffinity = −0.473 kcal/mol (Figure S2a,b). This variant was thus likely to disrupt normal protein–protein interactions. All in silico models similarly predicted that p.Lys50Asn is deleterious (REVEL, MutationTaster, PolyPhen2, PROVEAN, and SIFT).

This variant was tentatively identified as a pathogenic variant (PS1 + PS2 + PM2 + PP3 + PP4) as per ACMG guidelines.

#### 3.3.2 In silico analysis of the c.811_814del (p.Asn271Serfs*10) variant

The heterozygous c.811_814del variant in exon 1 of the *SOX11* gene is a frameshift variant. Sanger sequencing revealed it to be a de novo variant, and it has not previously been recorded in public databases. The variant was predicted to be deleterious and disease‐causing by PROVEAN and MutationTaster, respectively. A conservation analysis indicated that this variant occurred at a residue that is highly conserved in multiple species. Unlike p.Lys50Asn, the p.Asn271Serfs*10 variant is located outside the HMG box (Figure S1a,b).

The p.Asn271Serfs*10 variant was predicted to introduce a premature stop codon by SWISS‐MODEL, resulting in the production of a truncated protein lacking >10% of the overall amino acid sequence of the overall protein. Such a variant would severely damage overall protein structure, resulting in an overall loss of function and driving disease phenotypes. This mutant protein is also likely to be unstable, and is consistent with a loss of *SOX11* gene function.

Based upon the above evidence and in light of ACMG guidelines, we considered the variant to be pathogenic (PVS1_Strong + PS2 + PM2).

## 4 DISCUSSION

### 4.1 *SOX11* variants cause sensorineural hearing loss in patients with CSS

Two SOX11 missense variants were first reported by Tsurusaki, Koshimizu, et al. (2014), who identified two patients with CSS‐related phenotypic features including facial dysmorphia, mild intellectual disability, hypertrichosis, microcephaly, growth delay, and hypoplastic fifth toenails, leading to the determination that these were pathogenic variants associated with the CSS9 disease subtype. A number of other SOX11 variants have since been identified, including two missense variants, one nonsense variant, and chromosome 2p25 deletions that were detected by Hempel et al. (2016). All of these reported patients exhibited CSS‐like malformations, and all but two exhibited microcephaly. The spectrum of SOX11 variant‐related CSS9 disease phenotypes has expanded further in recent years to include certain congenital kidney and urinary tract abnormalities, cardiac anomalies, cleft palate, and hip dysplasia (Khan et al., 2018; Neirijnck et al., 2018; Okamoto et al., 2018). Given the variability in these clinical phenotypes, it is clear that a diagnosis of CSS cannot be excluded due to the absence of any specific feature, and that further studies of CSS9 patients may identify further traits associated with this condition. Further details about the phenotypic characteristics of these patients are shown in Table S1.

Herein, we reported two Chinese patients carrying two de novo (c.148A>C:p.Lys50Asn; c.811_814del:p.Asn271Serfs*10) variants in *SOX11*. In both cases, the patients exhibited phenotypes that were similar to those of other CSS patients with *SOX11* variants, including intellectual disability, dysmorphic facial features, growth delay, and hypoplastic fifth toenails. After clinical and molecular analyses, both patients were diagnosed with CSS9.

Both probands in this study exhibited varying degrees of sensorineural hearing loss with inner ear malformations. Neither of them failed the newborn hearing screening at birth. CT scans of the temporal bone revealed that proband 1 exhibited hypoplasia of lateral semicircular canal with cochlear aperture narrowing, while proband 2 cochlear segmentation insufficiency type II with cochlear aperture narrowing. While a sensorineural hearing loss phenotype has previously been reported in one case of CSS in 2008, a molecular diagnosis was not made in that report (Kellermayer et al., 2007). Otitis media leading to conductive hearing loss has been reported in CSS patients with *SOX11* variants, but sensorineural hearing loss associated with inner ear malformations has not been recorded as a significant complication of CSS caused by *SOX11* variants.

Interestingly, the patient reported by Hemple et al. had the same amino acid alterations as the proband 1, and both probands exhibited stunted growth and deformities of the fifth finger, with similar facial changes including flattened cheekbones and abnormally external genitalia. However, our patient exhibited sensorineural hearing loss, whereas their previously reported patient affected by conductive hearing loss. We speculate that the conductive hearing loss reported in their case may be caused by otitis media, as in most other CSS patients. SOX11 also exhibits significant functional redundancy, and disrupting its regulatory pathways may result in haploinsufficiency phenotypes in CSS patients (Turan et al., 2019), potentially explaining the observed phenotypic variability. In addition, we cannot exclude the possibility that these same variants may result in different phenotypes in different individuals.

A recent case with complex mosaic gain in the 2p25 region, which includes the *SOX11* gene, was diagnosed as CHARGE syndrome (Sperry et al., 2016), who exhibited hearing loss and inner ear deformities. Although the patients in the present study also exhibited growth and developmental delays, neither exhibited two or more of the classical diagnostic criteria associated with CHARGE syndrome (coloboma, choanal atresia, and semicircular canal anomalies), and as such, they were not diagnosed with this condition.

It is worth noting that proband 2 underwent cochlear implantation in the right ear at the request of the parents and received appropriate postoperative speech and language rehabilitation. This was despite the fact that hearing thresholds in both right ears were improved (Figure S3). Unfortunately, from a behavioral point of view, he did not achieve speech and language development comparable to hearing, and his CAP score improved only from 0 to 1. We conclude that his speech impairment was not only related to his hearing loss, but also to his intellectual development.

### 4.2 Molecular pathogenesis of *SOX11* variants

The human *SOX11* gene is located on chromosome 2p25 and encodes a member of the SOXC transcription factor family. The SOX11 protein contains a highly conserved N‐terminal HMG box DNA‐binding domain that mediates sequence‐specific DNA binding and a trans‐activating C‐terminal domain (Dy et al., 2008). SOX11 can facilitate chromatin opening, resulting in nucleolar remodeling and subsequent transcription (Dodonova et al., 2020). SOX11 expression is evident in a range of progenitor and stem cells wherein it controls their survival and fate determination in the context of neurogenesis, bone, kidney, and cardiac outflow tract formation (Hyodo‐Miura et al., 2002; Jia et al., 2020; Kavyanifar et al., 2018; Lefebvre, 2019; Lefebvre & Bhattaram, 2016; Paul et al., 2014; Sock et al., 2004; Wang et al., 2013; Yu et al., 2020). SOX11 serves as a transcriptional regulator, and the loss of its functionality can alter downstream target gene expression. Given the *SOX11* targets myriad genes, it is perhaps unsurprising that *SOX11* variants can result in a wide array of phenotypic differences.

*SOX11* gene is expressed at high levels in the context of inner ear development, and it is widely expressed in early ear vesicles. In animal model experiments, *SOX11* functionality has been shown to be integral to appropriate vestibular and auditory receptor development (Gnedeva & Hudspeth, 2015; Sinkkonen et al., 2011), potentially controlling the differentiation of hair cells by functioning downstream of the transcription factor Atoh1, which is required for the production of hair cells from the sensory epithelium of the inner ear (Chen et al., 2002; Helms et al., 2000). The results of the present study suggest that SOX11 is similarly involved in inner ear and auditory neuron development in humans.

There are certain limitations to the present study, as we did not assess the functions of cells from analyzed probands nor did we provide direct biochemical evidence validating our predicted structural models when evaluating the pathogenicity of variants of interest. In the future, we will seek to identify additional *SOX11* variant‐positive individuals and to construct knock‐in mice or other reliable functional models to further validate the spectrum of *SOX11*‐related disease phenotypes and to explore the underlying pathogenic mechanisms.

## 5 CONCLUSIONS

Herein, we utilized WES to identify two novel *SOX11* pathogenic variants associated with the development of CSS in two unrelated Chinese patients. Both patients are affected by sensorineural hearing loss with varying degrees of inner ear malformation, suggesting that *SOX11* may play a role in inner ear development. Our results extend the known spectrum of *SOX11* variants as well as the known spectrum of CSS9 phenotypic features. We recommend that genetic testing be performed as an important facet of the diagnosis and treatment of patients affecting by syndromic hearing loss.

## AUTHOR CONTRIBUTIONS

Yongyi Yuan and Pu Dai participated in the design of the study. Qiuquan Wang, Jie Wu, and Yongyi Yuan drafted the manuscript. Qiuquan Wang, Jie Wu, Jinyuan Yang, and Shasha Huang participated in data collection and data analysis. All authors read and approved the final manuscript.

## FUNDING INFORMATION

This study was supported by grants from National Key Research and Development Project of China (2016YFC1000706) and National Natural Science Foundation of China (81873704) and Fostering Funds of Chinese PLA General Hospital for National Distinguished Young Scholar Science Fund (2017‐JQPY‐001) to Yongyi Yuan，National Natural Science Foundation of China (81570929) to Xue Gao, National Key Research and Development Project (2016YFC1000700, 2016YFC1000704) and National Natural Science Foundation of China (81730029) to Pu Dai, National Institute on Deafness and other Communication Disorders (RO1 DC006483 and RO1 DC014496) to Xi Lin. The funders had no role in the study design, data collection and analysis, decision to publish, or preparation of the manuscript.

## CONFLICT OF INTEREST

The authors declare no potential conflicts of interest with respect to the authorship and/or publication of this article.

## Supporting information


**Appendix S1** Supporting Information Figures.

**Appendix S2** Supporting Information Tables.

## Data Availability

Data sharing is not applicable to this article as no new data were created or analyzed in this study.
