# Supplementary material for: Two SOX11 variants cause Coffin–Siris syndrome with a new feature of sensorineural hearing loss
Source: Am J Med Genet A. 2022 Nov 11;191(1):183–9. doi: 10.1002/ajmg.a.63011 (PMC10100107; doi:10.1002/ajmg.a.63011)
Supplement: Supplementary file 1 — Appendix S1 Supporting Information Figures. [file AJMG-191-183-s001.pdf]

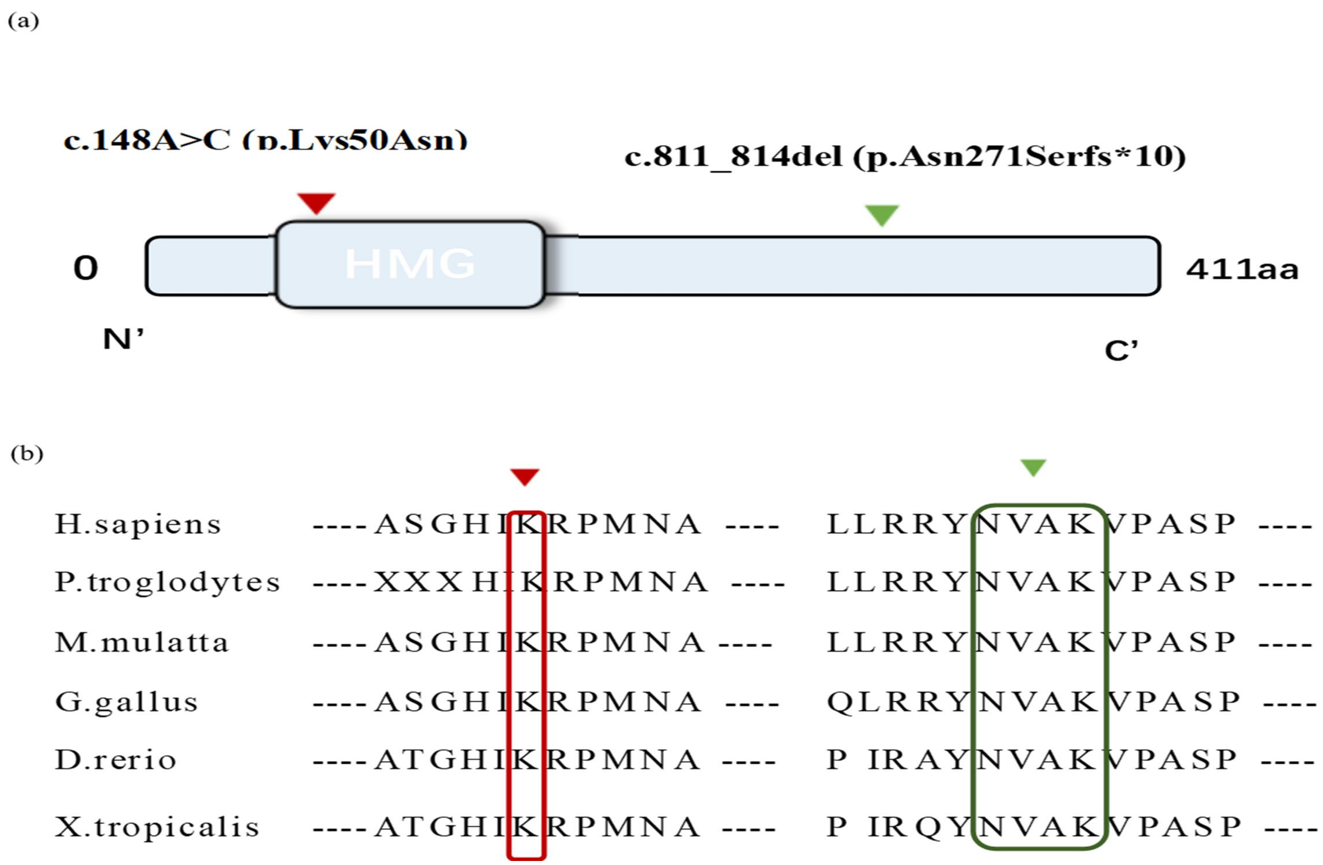

Supplemental Fig 1: Schematic representation of the<i> SOX11</i> gene including the location of the variants identified in proband 1 and 2. (a) The missense variant c.148A>C (p.Lys50Asn) identified in proband 1 was located in the HMG domain, while the frameshift variant c.811_814del (p.Asn271Serfs*10) identified in proband 2 resides outside the HMG domain. (b) Both the c.148A>C(p.Lys50Asn)(red box) and c.811_814del (p.Asn271Serfs*10)(green box) variants occur at evolutionarily conserved amino acids.

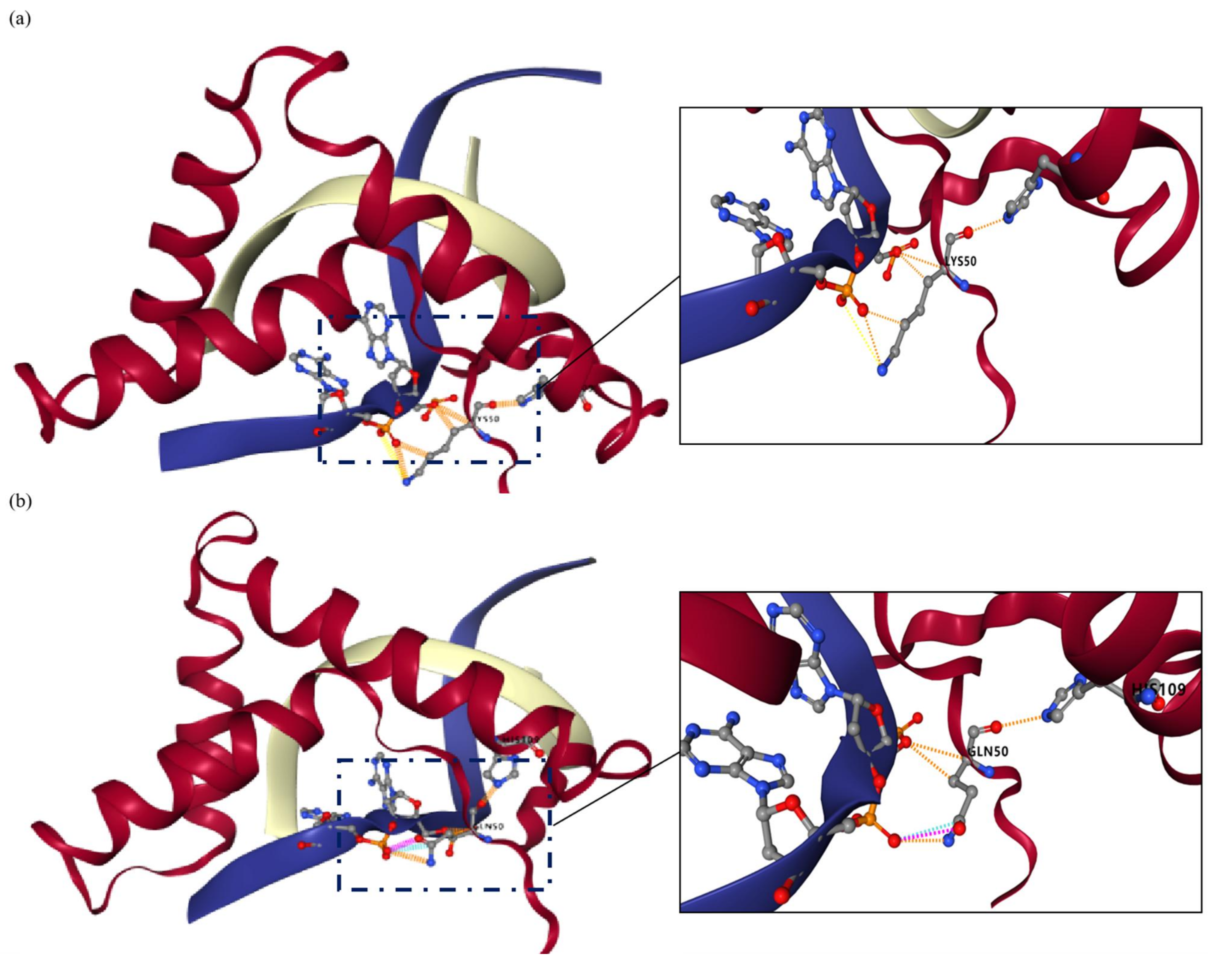

Supplemental Fig 2: Models of the structural changes in the SOX11 protein. (a) Wild-type SOX11. (b) Structures associated with disease-related amino acid substitutions. The c.148A>C variant caused the replacement of Lysine with Asparagine (p.Lys50Asn). The p.Asn 50 is involved in Van der Waals forces (shown as blue dotted lines) and clashes (shown as pink dotted lines) with adjacent amino acid residues. This missense variant was predicted to change the molecular structure and to interfere with DNA binding.

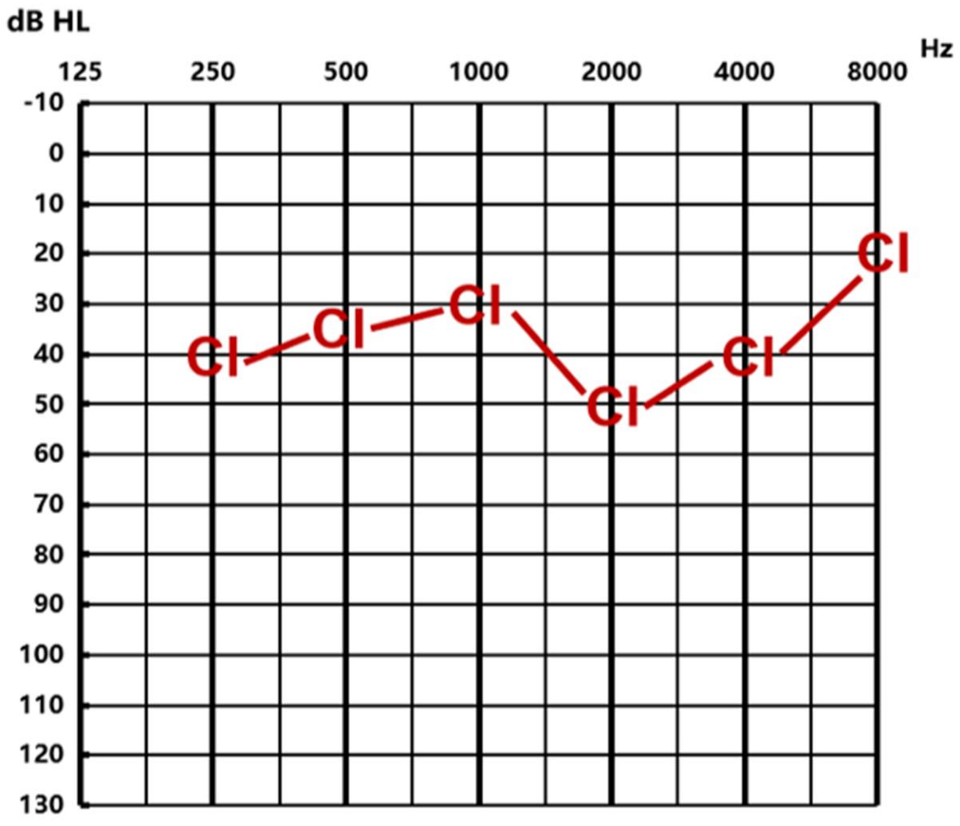

Supplemental Fig3: The right ear hearing threshold of proband 2 after cochlear implantation one year, CI means hearing threshold with cochlear implant.

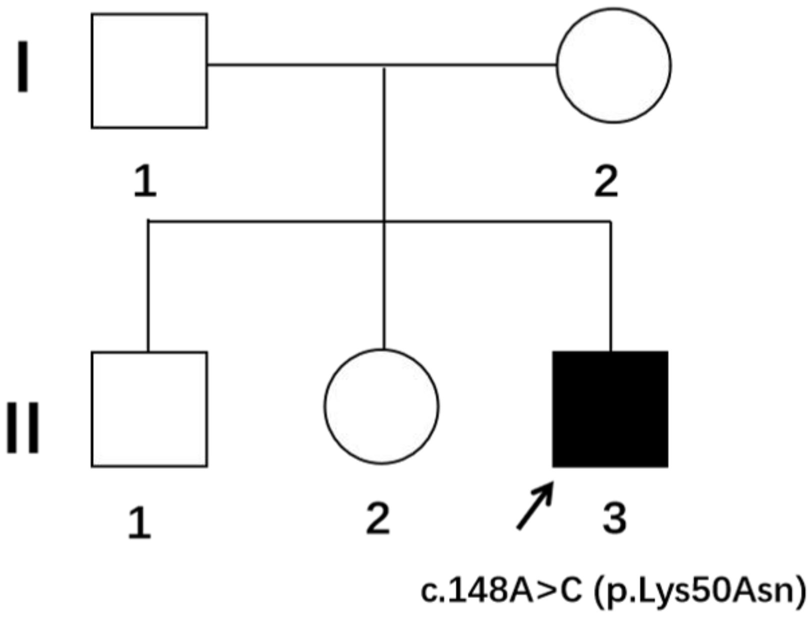

Supplemental Fig4: Pedigree of the family of proband 1.

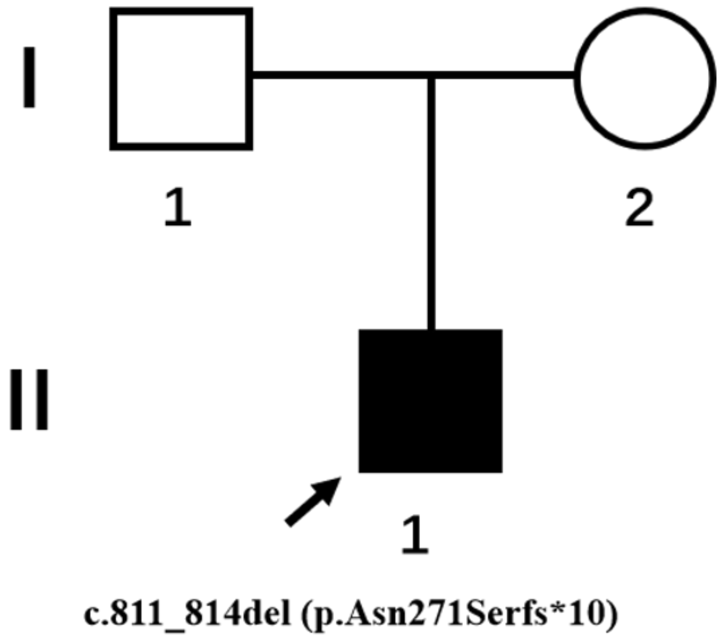

Supplemental Fig5: Pedigree of the family of proband 2.

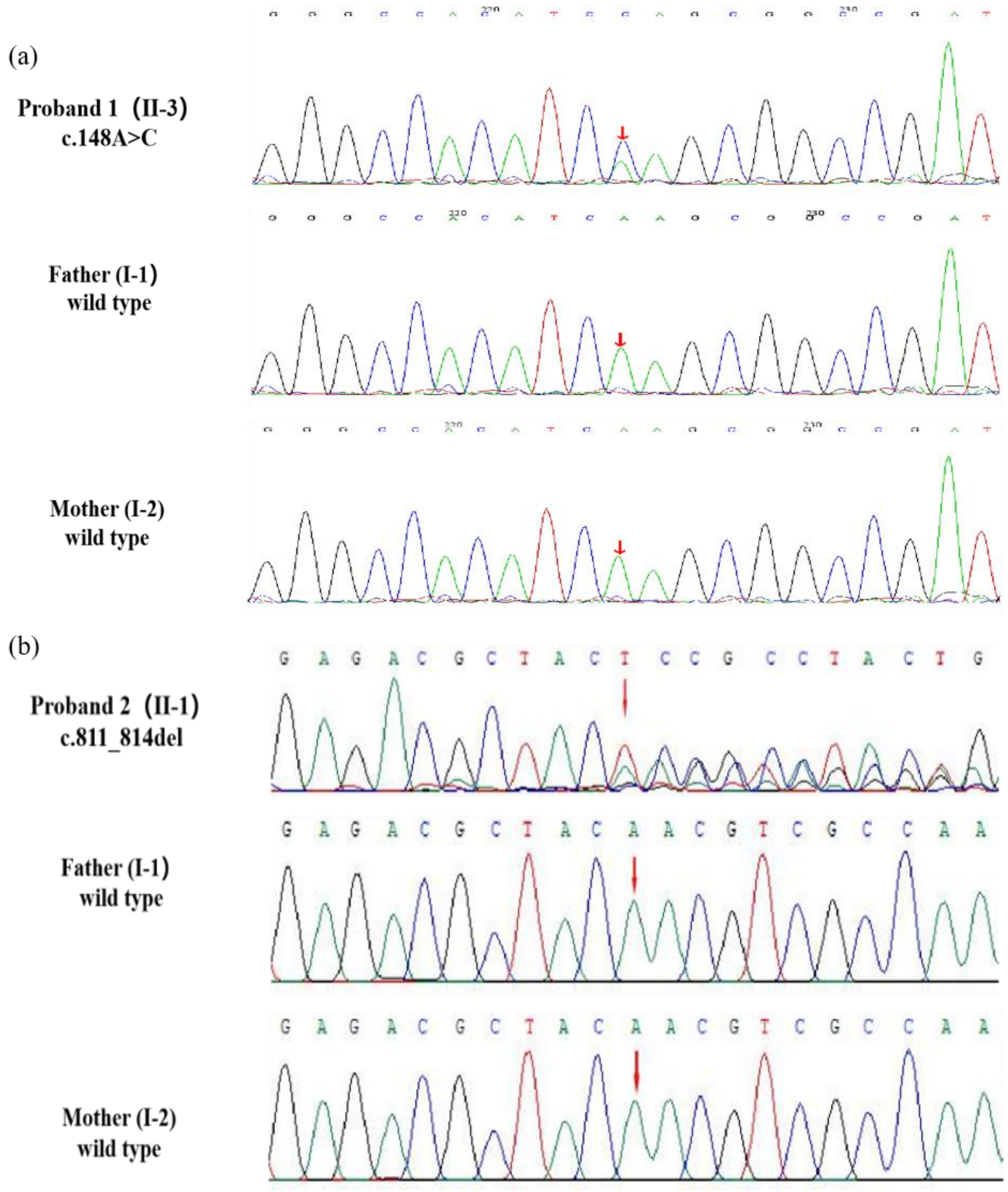

Supplemental Fig6: Sanger sequencing of proband 1 and proband 2.
(a)Sanger sequencing of proband 1 (II-3) and his parents (I-1,I-2).
(b)Sanger sequencing of proband 2 (II-1) and his parents (I-1,I-2).
