# Supplementary material for: Two SOX11 variants cause Coffin–Siris syndrome with a new feature of sensorineural hearing loss
Source: Am J Med Genet A. 2022 Nov 11;191(1):183–9. doi: 10.1002/ajmg.a.63011 (PMC10100107; doi:10.1002/ajmg.a.63011)
Supplement: Supplementary file 2 — Appendix S2 Supporting Information Tables. [file AJMG-191-183-s002.pdf]

Table 1. Review of All Cases of *SOX11* Variants and Coffin-Siris Syndrome

Clinical features	Tsurusaki et al.(2014)		Hempel et al.(2016)										Sperry et al.(2016)	Neirijnck et al.(2018)						Okamoto et al.(2018)	Khan et al.(2018)	Sekiguchi et al.(2019)		Timberlake et al.(2019)	This study	
	case1	case2	case3	case4	case5	case6	case7	case8	case9	case10	case11	case12	case13	case14	case15	case16	case17	case18	case19	case20	case21	case22	case23	case24	case25	case26
**Variant**	c.347A>G （p.Tyr116Cys）	c.178T>C （p.Ser60Pro）	c.359C>A （p.Pro120His）	c.150G>C （p.Lys50Asn）	c.87C>A （p.Cys29*）	2p25(4291420–6905655)del	2p25(5511851–16027633)del	2p25(5838893–7023548)del	2p25(5535091–16398225)del	2p25(2231163–300707)del	2p25(5209876–8078809)del	2p25(5166592–6400126)del	2p25(22572809-23174546)gain	c.63G>T （p.Thr21Thr）	c.257G>T （p.Arg86Leu）	c.658G>A （p.Val220Met）	c.1131C>T （p.Ser377Ser）	c.995C>T (p.Ser332Leu)	c.1063_1064insAGCGG CAGCAGC (p.S355-S356insSGSS)	c.305C>T (p.Ala102Val)	c.1148dupG (p.Gly384Argfs*14)	c.154C>T (p.Pro52Serb)	c.235A>G (p.Ile79Val)	c.191G>A (p.Arg64His)	c.148A>C (p.Lys50Asn)	c.811_814del (p.Asn271Serfs*10)
**Race**	Japanese	indian	English	English	English	English	English	English	English	English	English	English	American	Netherlands	Netherlands	Germany	Netherlands	Netherlands	Germany	Janpanese	English	Japanese	Japanese	American	Chinese	Chinese
**Gender**	Female	Female	Female	Male	Male	Female	Female	Female	Female	Male	Female	Female	Femal	Male	Male	Male	Male	Female	Male	Male	Male	NM	NM	Male	Male	Male
**Age at diagnosis (years)**	10years	17years	12years	11years	6years	12years	5years	14years	25years	34years	13years	2years	11years	NM	NM	NM	NM	NM	NM	NM	14years	NM	NM	NM	3years	10 months
**Gestation(weeks)**	38	NM	41	40	NM	41	36	37	NM	NM	NM	39	42	NM	NM	NM	NM	NM	NM	41	NM	NM	NM	NM	39	40.5
**Craniofacial anomalies**							NM				NM		-	NM	NM	NM	NM	NM	NM			NM	NM			
Coarse face (3/26)	-	-	-	-	+	NM		NM	NM	NM		NM	-							+	-			-	+	-
Mild facial hypoplasia(2/26)	+	-	-	-	-	NM		NM	NM	NM		NM	-							+	-			-	-	-
Frontal bossing/large forehead(4/26)	-	-	-	-	-	NM		+	NM	NM		NM	-							-	+			-	+	+
High frontal hairlin(3/26)	-	-	-	-	+	NM		NM	NM	NM		NM	-							-	+			-	-	+
Arched eye brow(5/26)	+	+	-	-	+	NM		NM	NM	NM		NM	-							+	+			-	-	-
Deep-set eyes(3/26)	-	-	+	-	-	NM		NM	NM	NM		+	-							-	+			-	-	-
Thick eyebrows(1/26)	-	-	-	-	-	NM		NM	NM	NM		NM	-							-	+			-	-	-
Short palpebral fissures(1/26)	+	-	-	-	-	NM		NM	NM	NM		NM	-							-	-			-	-	-
Flat nasal bridge(4/26)	+	-	-	-	-	NM		NM	NM	NM		NM	-							-	+			-	+	+
Short nose(2/26)	+	-	-	-	-	NM		NM	NM	NM		NM	-							+	-			-	-	-
Anteverted nares(1/26)	-	-	-	-	-	NM		NM	NM	NM		NM	-							-	-			-	+	-
Thin vermilion of the upper lip(1/26)	-	-	-	-	-	NM		NM	NM	NM		NM	-							-	-			-	+	-
Wide mouth(4/26)	-	-	-	-	+	+		NM	+	NM		NM	-							-	-			-	-	+
Thick lips(7/26)	+	-	+	-	-	+		NM	+	NM		+	-							+	-			-	-	+
Malar flattening(3/26)	-	-	-	+	-	NM		NM	NM	NM		NM	-							-	+			-	+	-
Tented upper lip(1/26)	-	-	-	+	-	NM		NM	NM	NM		NM	-							-	-			-	-	-
Everted lower lip(3/26)	+	+	-	-	-	NM		NM	NM	NM		NM	-							-	-			+	-	-
Abnormal ears(7/26)	+	-	-	+	-	+		NM	NM	NM		NM	+							-	-			+	+	+
Hgh palate(2/26)	+	-	-	-	-	NM		NM	NM	NM		NM	+							-	-			-	-	-
Cleft palate(1/26)	-	-	-	-	-	NM		NM	NM	NM		NM	-							-	+			-	-	-
Short philtrum(4/26)	+	-	-	+	-	NM		NM	NM	+		NM	-							+	-			-	-	-
Prominent philtrum(1/26)	-	-	-	-	-	NM		NM	NM	NM		NM	-							-	-			-	+	-
Long philtrum(1/26)	-	-	-	-	-	NM		NM	NM	NM		NM	-							-	-			-	+	-
Ptosis(1/26)	+	-	-	-	-	NM		NM	NM	NM		NM	-							-	-			-	-	-
**Neurodevelopment**																						NM	NM			
Development delay(10/26)	+	+(mild)	+	+	+(mild)	-	-		-	NM	-	-	+							+	+			+	+	+ (moderate)
Intellectual disability(15/26)	DQ:57	IQ:70-80	+(profound)	-	-	non-verbal IQ:54	NM	NM	+(severe)	+	-	-	-							DQ:40 (modeerate)	+			-	DQ:56	DQ:59
Seizures(2/26)	-	-	+	-	-	-	NM	NM	-	+	+	-	+							-	-			-	-	+
Hypotonia(5/26)	+	+	-	-	-	-	NM	NM	-	NM		-	-							-	+			-	+	+
Microcephaly(6/26)	+	+	+	+	-	-	NM	NM	-	NM	NM	+	+							+	+			-	-	-
Vision problems(7/26)	+	-	+	+	+	+	+	NM	+	NM	NM	-	+	-	-	-	+	-	+	-	-			+	-	-
Hearing anomalies(3/26)	-	-	-	bilateral conductive hearing loss	-	-	-	NM	-	NM	NM	-	right-sided profound sensorineural hearing loss; Mondini malformation; expanded endolymphatic duct	-	-	-	+	-	-	-	middle ear fluid			-	profound sensorineural hearing loss(right); severe sensorineural hearing loss(right); inner ear malformation	bilateral profound sensorineural hearing loss; inner ear malformation
Speech delay(7/26)	-	-	+	+	+	+	+	NM	+	+	+	+	+	NM	NM	NM	NM	NM	NM	+	+			-	+	+
Fine motor delay(1/26)	-	-	-	-	-	+	-	+	-	NM	NM	-	-	NM	NM	NM	NM	NM	NM		+			-	-	-
Poor weight gain(4/26)	-	-	+	+	-	-	+	NM	-	NM	NM	+	+	NM	NM	NM	NM	NM	NM	+	-			-	-	+
Hort stature (5/26)	+	+	+	-	-	-	+	NM	-	NM	NM	-	+	NM	NM	NM	NM	NM	NM	+	-			-	-	+
Behavioral abnormalities	+	-	autism;bruxism; tendency to pick skin	aggressive ; poor attention;no sense of danger	over-excitable behavior	autism; repetitive stereotyped movements with hyperactivity	-	NM	aggressive behaviour	NM	NM	-	-	NM	NM	NM	NM	NM	NM	hyperactive; aggressive	attention deficit; hyperactivity disorder; poor sleep			-	-	-
Walking delay	+	-	+	+	+	+	-	+	+	NM	NM	+	+	NM	NM	NM	NM	NM	NM	+	+	NM	NM	-	+	-
**Ectodermal**						NM			NM	NM	NM		-	NM	NM	NM	NM	NM	NM							
Hypoplastic fifth finger/toe nail(8/26)	+	+	-	-	+		NM	NM				+								+	+			-	+	+
Sparse scalp hair(5/26)	-	+(mild)	-	-	-		+	NM				+	-								+			-	-	+
Hypertrichosis(3/26)	+	+	-	-	-		NM	NM				-	-							+	-			-	-	-
Prominent eyelashes(1/26)	+	-	-	-	-		NM	NM				-	-							-	-			-	-	-
Abnormal/Delayed dentition(3/26)	+	-	-	-	-		NM	NM					+							-	-			-	+	-
**Skeletal**											NM	-	NM	NM	NM	NM	NM	NM	NM			NM	NM			
Aabsent/hypoplastic fifth finger/toenails(6/26)	-	+	-	-	-	-	NM	NM	+	NM		-								-	+			+	+	+
Clinodactyly(14/26)	+	+	+	+	+	+	+	+	-	NM		+								+	+			+	+	+
Syndactyly(4/26)	-	-	-	+	-	+	NM	+	+	NM		-								-	-			-	-	-
Delayed bone age(2/26)	-	-	-	-	-	-	NM	NM	-	NM		-								-	-			-	+	+
Scoliosis (4/8)						+	NM	+	+	+		-														
Craniosynostosis(1/26)	-	-	-	-	-	-	NM	NM	-	-		-								-	-			+	-	-
**Gastrointestinal**														NM	NM	NM	NM	NM	NM			NM	NM			
Feeding difficult(8/26)	+	-	+	+	-	-	NM	+	-	-	+	+	NM							-	+			-	-	+
Sucking difficule(3/26)	+	-	-	+	-	-	NM	+	-	-	NM	-	-							-	-			-	-	-
Gastro-oesophageal reflux (3/8)						+	+	NM	-	-	NM	+	-													
**Others**							NM	NM	-	-	NM	NM										NM	NM			
Antenatal anomalies	IUGR	IUGR	-	third trimester bleeding	-	fetal bradycardia; oligohydramnios			reduced fetal movements	-	NM	NM	-	-	-	-	-	-	-	-	-			-	-	fetal nasal bone loss
Renal abnormalities	left small kidney	bilateral malrotated kidneys	-	-	-	-			-	-	NM	NM	bilateral renal calculi and cysts	posterior urethral valves	multicystic / kidney dysplasia (left)	posterior urethral valves; kidney hypoplasia (bilateral)	ureteropelvic junction obstruction (left)	duplex collecting system; Ureteropelvic junction obstruction; Vesicoureteralreflux (right)	posterior urethral valve; multicystic kidney dysplasia (bilateral)	-	-			-	-	-
Genital anomalies	-	hypoplastic uterus; hypogonadotrophic hypogonadism	-	cryptorchidism	-	-			-	cryptorchidism	NM	NM	hypoplastic labia minora	-	-	-	maldescended testis (right); urethral meatal stenosis	-	-	-	-			-	small external genitalia	-
Cardiac anomalies	-	kidneys.	-	-	-	-			-	-	NM	NM	-	-	-	-	-	-	-	coarctation of the aorta; VSD	-			-	-	-
Frequent infections						-			persistent ductus arteriosus	-	NM	NM	-													
Choanal stenosis						-			+	-	NM	NM	-													
Inverted nipples						+		+	-	-	NM	NM	-													

velopment quotient; IUGR:intrauterine growth restriction; NM: Not Mentioned

phenotypes, there are some rare phenotypes that are not marked, such as short jaws, gastritis
